# Building an *ab initio* solvated DNA model using Euclidean neural networks

**DOI:** 10.1371/journal.pone.0297502

**Published:** 2024-02-15

**Authors:** Alex J. Lee, Joshua A. Rackers, Shivesh Pathak, William P. Bricker

**Affiliations:** 1 Department of Chemical and Biological Engineering, University of New Mexico, Albuquerque, NM, United States of America; 2 Center for Computing Research, Sandia National Laboratories, Albuquerque, NM, United States of America; Shiv Nadar University, INDIA

## Abstract

Accurately modeling large biomolecules such as DNA from first principles is fundamentally challenging due to the steep computational scaling of *ab initio* quantum chemistry methods. This limitation becomes even more prominent when modeling biomolecules in solution due to the need to include large numbers of solvent molecules. We present a machine-learned electron density model based on a Euclidean neural network framework that includes a built-in understanding of equivariance to model explicitly solvated double-stranded DNA. By training the machine learning model using molecular fragments that sample the key DNA and solvent interactions, we show that the model predicts electron densities of arbitrary systems of solvated DNA accurately, resolves polarization effects that are neglected by classical force fields, and captures the physics of the DNA-solvent interaction at the *ab initio* level.

## Introduction

The explosion in the use and development of machine learning (ML) methods to solve scientific problems has spread to and become firmly entrenched in the field of quantum molecular modeling. One of the key benefits of machine learning approaches in this realm is that restrictive computational scaling limitations stemming from the cost of solving Schrödinger’s equation can be bypassed. This opens up the possibility of studying large biological macromolecules such as DNA, for which the broad application of quantum modeling has been limited by prohibitively large system sizes [1]. To date, machine learning methods have been applied to predict accurate potential energy surfaces and forces [2–8] and electron densities [9–17] of mostly small molecules and crystals. For DNA specifically, a machine learning method has been used to develop a density functional theory (DFT) functional that correctly describes charge delocalization in base pairing [18].

DNA makes an excellent test system for building a prototypical machine learning model for a biological macromolecule. Compared to a protein, the DNA double helix has a predictable structure with considerably less conformational variation due to strict base pairing rules [19, 20], making DNA structural information easier to sample. Furthermore, DNA can be easily split into a small number of component parts that make for suitable training structures, consisting of only the four nucleobases (A, C, T, and G), sugar rings, and phosphate groups. With these considerations, we previously constructed a machine learning model that computes *ab initio* electron densities for DNA structures of arbitrary size, tested for systems with up to 20,000 atoms [21]. However, this previous model only accounted for DNA by itself (gas-phase DNA) without the inclusion of solvent.

Of course, for most practical applications, *in vivo* or *in vitro*, DNA exists in an aqueous environment. Further, DNA’s interactions with its solvent environment are crucial for influencing its properties [22]. Therefore, we extend our previously developed gas-phase DNA model to describe solvated DNA with the DNA-solvent interactions modeled explicitly. Machine learning is well-suited to this task for several reasons. First, the main challenge with including explicit solvent in the model is that system sizes quickly become computationally intractable due to the sheer numbers of individual solvent molecules that need to be included. Machine learning models have been shown to break through traditional scaling limits and can handle large-scale systems that are well-outside the bounds of conventional methods [12, 17, 21]. Second, density-based machine learning models can accurately describe the prominent types of interactions for solvated DNA systems. Our previous gas-phase DNA model showed that internal DNA interactions can be accounted for [21], and other studies have been able to capture non-covalent interactions [10, 23], which are the dominant type of interaction between DNA and solvent.

Solvent interactions have also been shown to be important in DNA binding studies. In non-contact or “indirect readout” binding, a binding agent targets specific segments of DNA without directly reading in the base sequence [24–26]. Instead, the binding agent recognizes the target based on properties such as the electrostatic potential that can be influenced by the base sequence. An example of this are DNA systems with A-tracts (base sequence segments with four or more consecutive A bases) which exhibit unique structural features, including intensified bending of the helix as well as narrowed minor grooves [27]. The narrowness of the minor groove effectuates an enhanced negative electrostatic potential around the A-tract sequence, which is thought to be the mechanism for site-specific binding of many minor-groove binding proteins [24–26, 28]. This phenomenon has been studied with coarse-grained implicit solvent models, where the enhancement of the electrostatic potential is shown to be driven by solvation [24–26]. An explicit solvent model that could study solvation effects at the *ab initio* level of theory would thus be useful for studying these phenomena in greater detail.

In order to extend the previous gas-phase DNA machine learning model to account for explicit solvent interactions, modifications to the training procedure were necessary. To accommodate the solvent molecules into the training set, the fundamental base-pair step training structures for duplex DNA were split into smaller overlapping fragments that sample the key interactions for a solvated DNA system. By comparing the predictions of machine learning models trained differently, we gain additional insight into how machine learning models learn and make predictions from their training data. Another possible benefit from using smaller training fragments is that training on larger basis sets and higher levels of theory becomes more computationally tractable. It has been shown that larger basis sets are needed to calculate accurate forces from electron densities [29]. The efficient calculation of forces could be useful for structural optimization and *ab initio* molecular dynamics simulations, leading to potential applications in structural prediction and biomolecular binding studies.

## Results and discussion

### Model training overview

The procedure for training the DNA-solvent machine learning model is similar to that used in the previous gas-phase DNA model [21, 30]. We provide an abbreviated description of the procedure here, focusing on the modifications that were performed to adapt the training procedure to the current model. The training procedure is outlined below, with full details provided in Computational Methods:

- The fundamental training unit for the model is the B-DNA base-pair step (two adjacent base pairs). Accounting for symmetry, there are ten total unique combinations of base-pair steps.
- All-atom molecular dynamics simulations are run to obtain configurational snapshots for each of the DNA base-pair step training units in solution.
- The training units are further broken up into smaller overlapping fragments that sample the key interactions. These fragments include DNA only, solvent only, and DNA with explicit solvent.
- *Ab initio* calculations using density functional theory are run on the DNA-solvent fragments to compute ground-state electron densities. The basis set coefficients for the electron densities are the specific data used to train the machine learning model.
- The fragment density data are used to train a graph convolutional neural network model. A trained model can take as input any arbitrary solvated DNA structure and output an electron density without performing a traditional (and often computationally costly) quantum calculation.

The B-DNA base-pair step was chosen as the fundamental training unit for the model because it is the smallest structural unit that captures three key DNA interactions: the hydrogen bonds between complementary base pairs (A/T and G/C) that hybridize the two strands, the base stacking interactions between adjacent bases that stabilize the DNA double helical structure, and the covalent bonds between nucleotide components that form the DNA backbone. DNA-solvent interactions are sampled by including explicit water molecules around the DNA base-pair step.

### Fragmentation procedure for the DNA-solvent training set

**Fig 1:**
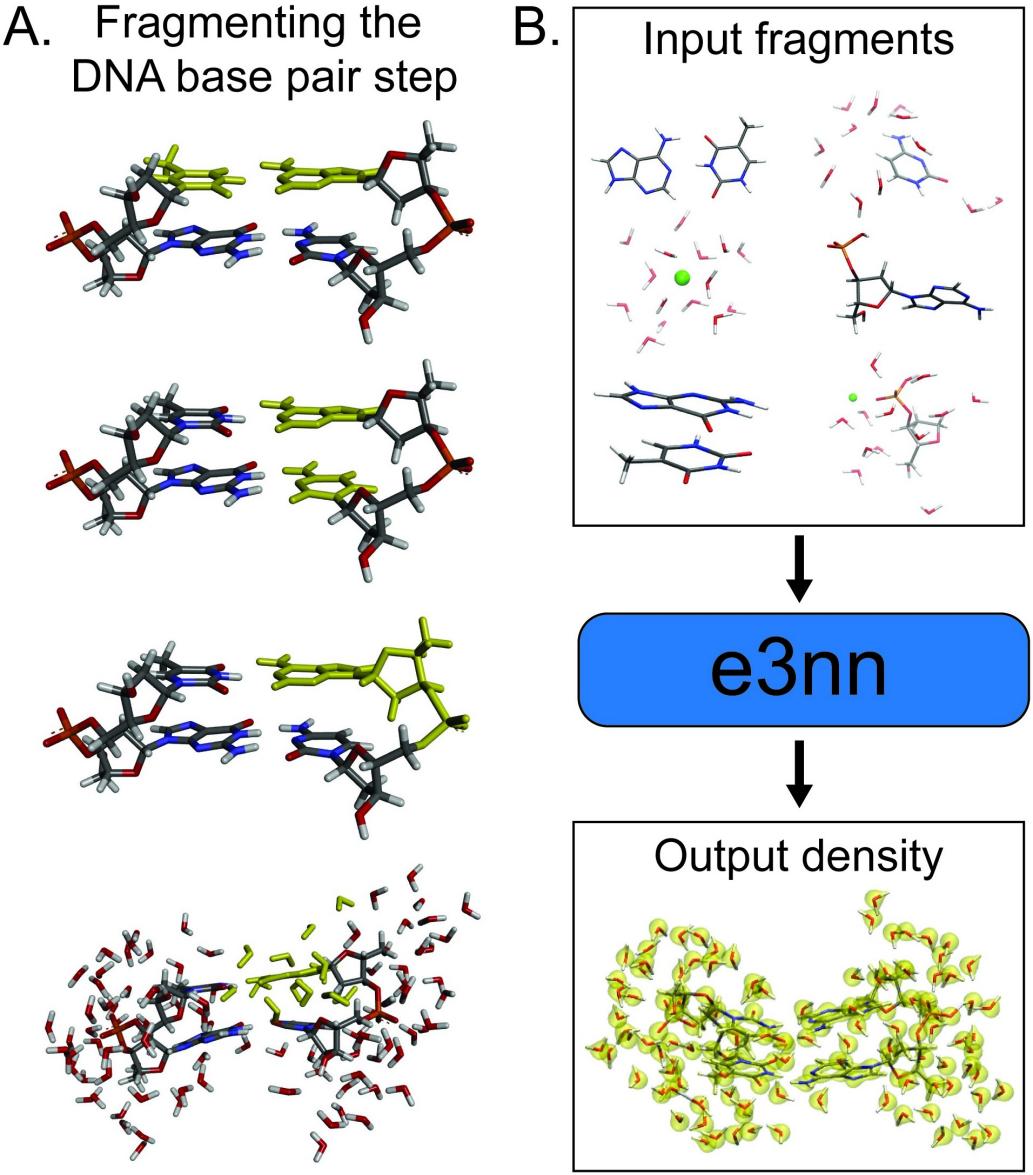
Schematic for training the machine learning model using a fragmentation procedure. (A) Breaking the fundamental DNA base-pair step unit down into smaller training fragments. From top to bottom, fragments are highlighted in yellow for DNA base pairs, base stackings, nucleotides, and DNA base and solvent. (B) Fragmented training structures sampled from MD simulations are used to train an e3nn neural network model. A trained model outputs electron densities for arbitrary solvated DNA structures. Figures were made with Discovery Studio Visualizer [32] and VMD [33, 34].

The fundamental base-pair step training structures are broken into smaller overlapping fragments that are placed into three categories: DNA only, solvent only, and DNA with solvent. For the first set which includes only DNA, we ensure that the fragmented training structures retain the key double-stranded DNA interactions by including separate fragments for base pairing, base stacking, and nucleotides (Fig 1A). The second set of structures, solvent only, includes ion solvation shells for Mg^2+^ and Cl^-^ surrounded by 15 waters (slightly over two water shells) in addition to 15 water only clusters taken from a database used for a previous water density model [17, 31]. The last set of structures, DNA with solvent, is broken into fragments for each of the DNA bases (A, C, G, and T) surrounded by 12 water molecules (approximately two water shells) as well as DNA backbone sugar-phosphate fragments surrounded by 12 waters. Fragments with Mg^2+^ bound to the phosphate are sampled by including structures where the Mg-O atomic distance is less than 2 Å. All dangling bonds in the fragments are capped with H atoms.

We note that the training structures used in this study do not represent the only possible way to construct fragments for a DNA model. As a guiding principle, we aimed for fragment sizes of 30 to 60 atoms, which is two to four times smaller than the previous model’s training structures with the entire base-pair step [21, 30]. Further, we constructed the fragments such that the key local interactions (covalent and non-covalent) for a double-stranded DNA system in solution are sampled by the training set. The contents of the training set are summarized in Table 1, and a full description of training set contents are reported in the Supporting Information in S1–S3 Figs. A schematic for the training procedure is shown in Fig 1B.

**Table 1:** Summary of the DNA-solvent model training set.

Structure	Fragments	Samples
DNA only	8*10	16,000
Solvent only	3	1,600
DNA with solvent	6	1,800
Total		19,400

### Accuracy of electron density predictions of a fragmented DNA only training set

**Fig 2:**
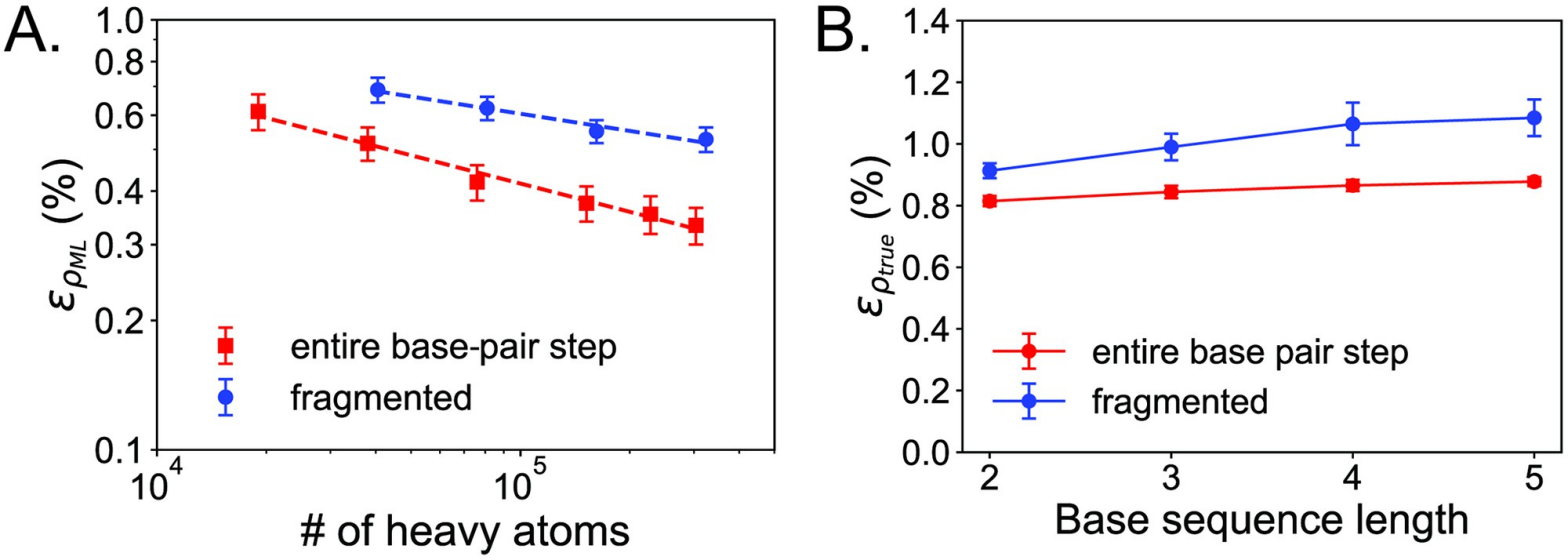
DNA only model prediction errors for electron density. (A) Learning curves on a log-log scale for increasing numbers of training samples for a test set of base-pair step (two base pairs) structures. For a fairer comparison between the models, we normalize to the number of heavy (non-H) atoms in the training data. (B) Errors ${∊}_{\rho_{true}}$ for test set structures with increasing base sequence lengths. Both models were trained with the largest set of training data, containing around 300,000 heavy atoms.

Prior to including explicit solvent, we first consider a model trained on DNA only data (Table 1) to assess the strategy of fragmenting the DNA base-pair step. We compare density prediction errors ${∊}_{\rho_{ML}}$ and ${∊}_{\rho_{true}}$ (see Computational Methods for definitions of these errors) from the current fragment-trained model and the previous model trained on the entire DNA base-pair step [21, 30]. Note that the accuracy of the previous model has been improved from what was reported [21] by normalizing the coefficients to electron populations (see S1 Appendix). Across a holdout test set that includes all possible combinations of base-pair step structures (for details on the test set structures see S5 and S6 Tables), we plot learning curves based on the number of heavy (non-H) atoms in the training data (Fig 2A). For both models, the learning curves are essentially linear on a log-log scale, which show that the models meaningfully learn from their training data [35].

As expected, compared to the previous model trained on the entire base-pair step, the fragment-trained model has larger density prediction errors (${∊}_{\rho_{ML}}\left(\%\right)$) and also learns less per heavy atom in the training set, judging from the shallower slope of its learning curve. This is not surprising because the fragmented training structures are represented in the network as smaller graphs with fewer connections and thus contain inherently less information for the model to learn. Despite the loss of accuracy, the fragment-trained model still produces highly accurate densities that are close to the state-of-the-art, with an error as low as ${∊}_{\rho_{ML}}=0.53\%$ for the model trained with 300,000 heavy atoms. Some of the current most accurate machine learning density models report errors as low as 0.3% for small molecules (less than 25 atoms) [10, 14, 16] ranging up to 2.5% for more complex molecules and small proteins [12]. We note that the DNA structures in our test set contain around 125 atoms and are considerably more complex than the small molecules tested in the above studies, yet the error of ${∊}_{\rho_{ML}}=0.33\%$ for the model trained on the entire base-pair step, and the error of ${∊}_{\rho_{ML}}=0.53\%$ for the model trained on DNA fragments both compare well with these studies. Furthermore, when testing on larger DNA structures, the fragment-trained model shows the same behavior as the previous model in that the error flattens out with progressively longer base sequences, approaching a value of around ${∊}_{\rho_{true}}=1.08\%$ (Fig 2B) for a DNA 5-mer. This suggests that both the fragmented and entire base-pair step models are suitable for making predictions on larger DNA sequences with a negligible loss of accuracy.

Two likely sources are responsible for the loss of accuracy when training on smaller fragments. The first is that smaller training structures inherently contain less information about long-range density correlations. Note that for any machine learning model that makes predictions on large-scale biological macromolecules such as DNA, the training structures will necessarily be smaller than the full system structures and thus naturally impose a cut-off in the long-range correlations that can be sampled by the training set. That is to say, there is an intrinsic trade-off between model accuracy and training fragment size, which affects the cost of training the model. For the current study, the fragment structures are two to four times smaller than the entire base-pair step structures, giving a model error increase from ${∊}_{\rho_{ML}}=0.33\%$ up to ${∊}_{\rho_{ML}}=0.53\%$. Because larger basis sets are required to calculate accurate forces [29] from training structures, the cost savings in using smaller training fragments may be worth the small increase in model error. The second source of error comes from artifacts caused by the fragmentation procedure itself. Recall that dangling bonds in training structures were capped with H atoms, which do not necessarily reflect the molecular or bonding environments of the full DNA system. This also implies that the more aggressive the fragmentation strategy, the more the artifacts of capping the fragments with H will contaminate the subsequent model predictions.

We can observe this by comparing the errors in the number of electrons predicted by the two models from the signed relative errors, ${∊}_{N_{ele}}=\left(N_{ele,ML}-N_{ele,true}\right)/N_{ele,true}$, where *N*_*ele*,*ML*_ are the number of electrons predicted by the machine learning model and *N*_*ele*,*true*_ are the reference number of electrons from *ab initio* calculations. While the models are not constrained to predict the number of electrons exactly, they still predict these values with high accuracy, giving errors of much less than one electron out of around 580 total in the test set structures. From the data in Table 2, the model trained on the entire base-pair step has a low signed relative error of 0.0036%, suggesting that the distribution of the errors is centered around the reference value, with positive and negative errors cancelling out. On the other hand, the signed mean error for the fragment-trained model is more negative with a greater magnitude (−0.020%), suggesting that the fragment-trained model systematically underestimates the number of electrons for a given test structure, although this underestimation is still very small compared to reference. The underestimation makes sense considering that capping the training fragments with H will contribute fewer electrons compared to the full DNA structures. The underestimation of electrons extends to the tests on longer DNA structures (S9 Table). Note that in this case, both models underestimate the number of electrons. However, the underestimation in the fragment-trained model is noticeably larger due to the more aggressive fragmentation. Thus, for the fragment-trained model in particular, it may be possible to improve model accuracy further by employing a charge equilibration scheme [36] to constrain the model to predict the correct number of electrons.

**Table 2:** DNA only model prediction errors for electron density and number of electrons. Errors are averaged across 300 test structures that include all ten possible combinations of base-pair steps. The table shows density prediction errors (*ϵ*_*ρ*_) and signed relative errors ${∊}_{N_{ele}}$ in the predicted number of electrons for each model.

Model	${∊}_{\rho_{ML}}\left(\%\right)$	${∊}_{\rho_{true}}\left(\%\right)$	${∊}_{N_{ele}}\left(\%\right)$
entire base-pair step	0.33±0.03	0.81±0.02	0.0036±0.028
fragmented	0.53±0.03	0.92±0.02	−0.020±0.049

### Polarization effects in a solvent only model

**Fig 3:**
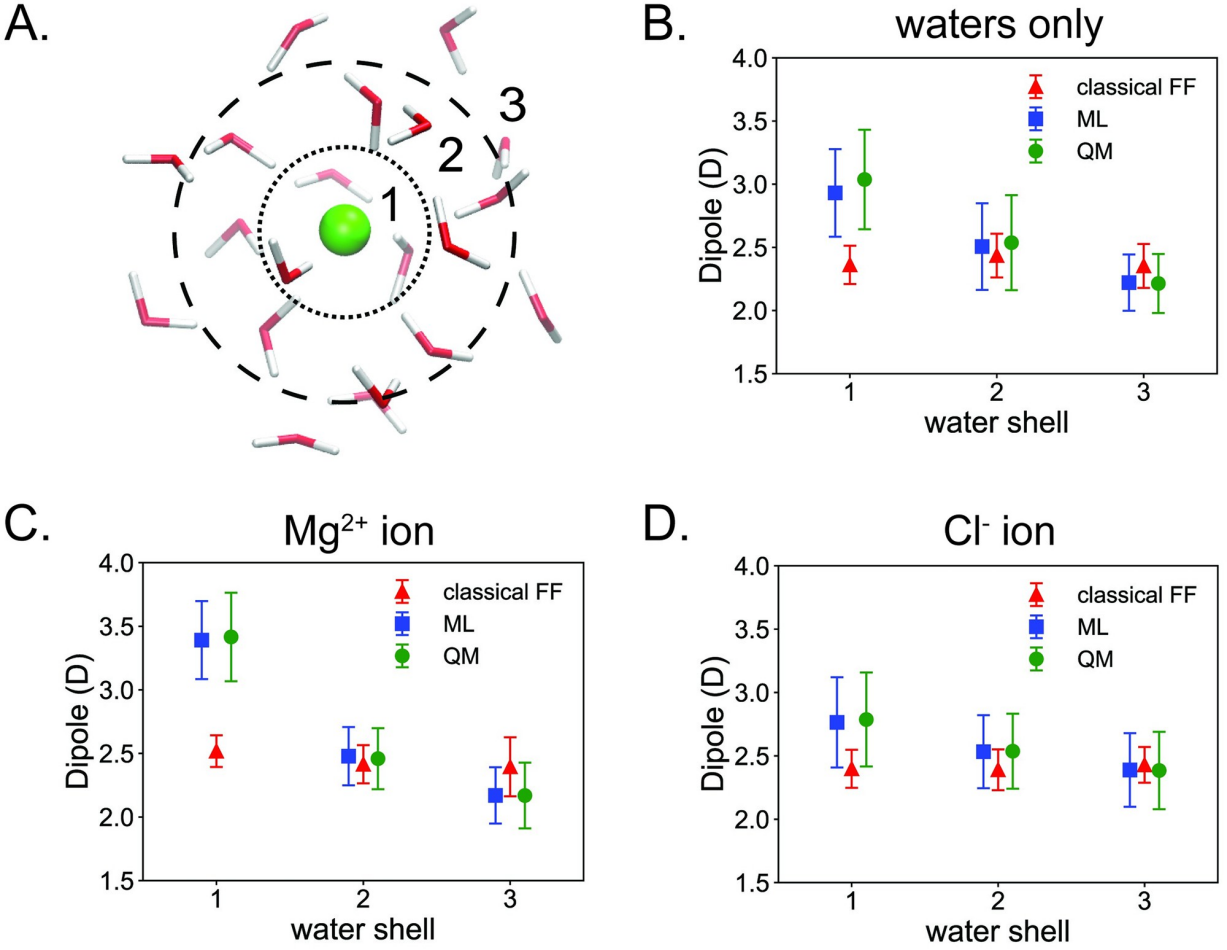
Polarization effects of a solvent only model. (A) Illustration of a solvated ion test structure. Waters are categorized into one of three water shells based on their distance from the ion. The graphic was made with VMD [33, 34]. Comparison of averaged dipole moments of water molecules within a water shell for test structures of (B) water only clusters, (C) solvated Mg^2+^, and (D) solvated Cl^-^ clusters (S2 Table). Taking *r* as the distance between the ion and the O atom in a water molecule, water shell 1 contains waters from 0 < *r* < = 3.4 Å, water shell 2 from 3.4 < *r* < = 5.0 Å, and water shell 3 from *r* > 5.0 Å. Each test structure contains 20 water molecules.

Next, we discuss a model trained on solvent only fragments (Table 1). In particular, we assess how well the model can reproduce solvent polarization effects that are neglected by statically parameterized classical force fields. We do this by calculating dipole moments of individual water molecules around ion solvation shells (Fig 3A) and comparing results from the model to a classical BSC1 force field [37, 38] and to reference quantum calculations.

The test set includes ten snapshots each of both of the ions in our MD simulations (Mg^2+^ and Cl^-^) surrounded by 20 water molecules as well as ten structures of 20 water only clusters with no ions (S7 Table). These test structures are slightly larger than the solvent data the model was trained on, which were water clusters consisting of 15 molecules [31]. Water molecules in the test structures are categorized into one of three water shells based on the distances of their O atoms from the ion (for the water only clusters, because there is no ion we use the distance from the center of nuclear charge for the cluster). Taking the default values from Amber’s CPPTRAJ analysis program [39], the first water shell is 0 < *r* < = 3.4 Å, the second water shell is 3.4 < *r* < = 5.0 Å, and the third shell contains the remaining waters *r* > 5.0 Å.

Dipole moments for individual water molecules were calculated by integrating their electron densities on a 0.1 Bohr cubic grid. These electron densities were partitioned by using only the output basis functions for the respective atoms in the water molecule. Throughout the analysis, water molecules may not be precisely neutral due to charge transfer, numerical error in integrating densities on the grid, and machine learning error, so calculated dipoles will be dependent on a reference point. We standardize our dipole calculations by choosing the reference point to be the center of nuclear charge for each water molecule.

First, we confirm that the solvent only model accurately predicts electron densities across the test set structures. The density errors are ${∊}_{\rho_{ML}}=0.35\pm 0.03$%, 0.34±0.03%, and 0.39±0.03% for the Mg^2+^, Cl^-^, and water only clusters, respectively, which are slightly more accurate than those from the DNA only model. For each of the test systems, Fig 3B–3D show the average dipole moments for the water molecules within each water shell. Notably, the classical force field produces dipole moments that are essentially the same for each water shell, resulting in a flat trend. This is not surprising since the classical force field calculates dipoles from statically parameterized partial atomic charges, so only the geometry of the water molecule will affect its dipole.

On the other hand, the machine learning model shows excellent agreement with the reference quantum calculations for all three test cases. The model correctly predicts that molecules in shells closer to the ions have larger dipole moments and thus are more strongly polarized. Further, the polarization effect is larger around Mg^2+^ compared to Cl^-^ because the ion is twice as charged. Note that the model tends to slightly underestimate the dipole moment. This underestimation is most likely related to the test clusters (20 molecules) being larger than the water clusters the model was trained on (15 molecules). It has been shown that polarization effects increase with water cluster size, approaching bulk water behavior in clusters with more than 26 water molecules [40, 41]. In addition, waters near the centers of clusters have been shown to be more strongly polarized compared to waters on the edges of clusters due to their greater numbers of hydrogen bonds [41]. Based on the water only data (Fig 3B), the machine learning model captures this behavior and also matches with quantum reference calculations, as waters near the center of the cluster (water shell 1) have dipole moments close to bulk water (2.9 D) whereas waters on the edges of the cluster (water shell 3) have dipole moments that are approaching monomeric water (1.85 D) [40, 41].

### Combined DNA-solvent electron density model predictions

**Fig 4:**
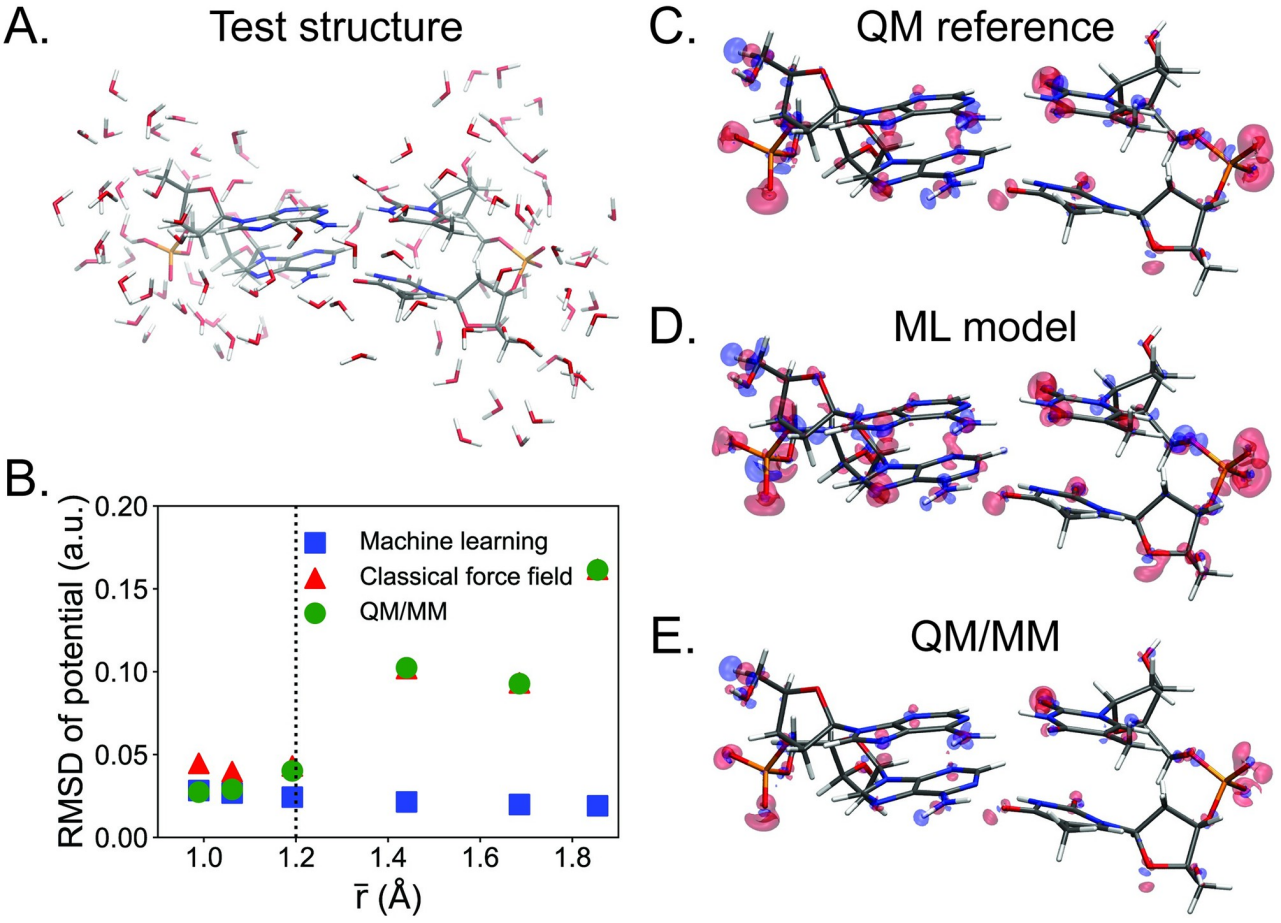
Assessing the DNA-solvent interaction with electrostatic potentials and density difference maps. (A) A representative static snapshot of a DNA-solvent test structure. (B) RMSD values of electrostatic potential surfaces against quantum reference calculations. The surfaces are characterized by $\bar{r}$, the average distance of the potential surface to the nearest DNA atom. To get a sense of the interaction range, the van der Waals radius for hydrogen is shown at *r* = 1.2 Å (dotted line). The nearest solvent atoms in the first solvation shell are around *r* = 1.4 Å from the DNA atoms. Density difference plots for (C) a reference quantum calculation, (D) the machine learning model, and (E) a QM/MM calculation, respectively. The plots are obtained by taking the “polarized” DNA density from the DNA-solvent system and subtracting out the DNA only density, *ρ*_*DNA*,*polarized*_ − *ρ*_*DNA*,*gas*_. Red and blue represent an excess and a deficiency of electron density on an isosurface plotted at ±0.005 a.u. Figures were made with VMD [33, 34].

Having shown that the fragmented training procedure produces accurate DNA only and solvent only electron density models, we now combine all of the training sets in Table 1 to construct a model for an explicitly solvated DNA system. The test structure for this model is a DNA base-pair step surrounded by 100 water molecules (Fig 4A) which is slightly more than a single water shell. We include ten test structures for each combination of base-pair step (ten combinations total), as well as ten structures with an Mg^2+^ bound to the phosphate for a total of 110 test structures (S8 Table), each of which contain around 480 atoms. Due to the size of the test structures, we omit calculating ${∊}_{\rho_{ML}}$, which requires an additional projection to the auxiliary density basis, and only calculate ${∊}_{\rho_{true}}$, noting that ${∊}_{\rho_{ML}}$, which is the error from fitting the model, will always be lower.

As a direct comparison, we trained an additional model that treated the H and O atoms in water molecules as distinct element types from the H and O atoms in DNA. Since DNA and solvent molecules are unambiguously identifiable, we were interested to determine if the added element types would increase the specificity of learning the chemical environments around atoms and improve the accuracy of the model. As detailed in Computational Methods, element types are encoded as input features in the neural network graphs. In the “distinct waters” model, the number of element types is increased from seven to nine (H, C, N, O, P, Mg, and Cl for the general model, plus HW and OW as distinct water molecule element types).

The results for both models are shown in Table 3. Across the test set, the general model predicts a density error of ${∊}_{\rho_{true}}=1.13+0.02\%$. While the error is low, it is larger than that of the DNA only model (${∊}_{\rho_{true}}=0.92\%$), reflecting the greater complexity of the solvated DNA system. Somewhat disappointingly, the distinct water model shows no improvement over the general model with an error of around ${∊}_{\rho_{true}}=1.17+0.02\%$. But on a positive note, this implies that the general model sufficiently distinguishes the chemical environments for DNA and water without needing distinct element types for DNA and water atoms. Therefore, the general model was used to obtain the remainder of the results in the study.

**Table 3:** Combined DNA-solvent model prediction errors for electron density and number of electrons. Errors are averaged across 110 test structures that include all ten unique combinations of base-pair steps each solvated with 100 water molecules, including structures with Mg^2+^ interacting with the phosphate group. The table shows the density prediction errors (*ϵ*_*ρ*_) and the signed relative errors ${∊}_{N_{ele}}$ in the predicted number of electrons for each model.

Model	${∊}_{\rho_{true}}\left(\%\right)$	${∊}_{N_{ele}}\left(\%\right)$
general	1.13 ± 0.02	0.063 ± 0.037
distinct waters	1.17 ± 0.02	0.0019 ± 0.044

To obtain a more physically meaningful sense of the density error, the electrostatic potential was calculated from the machine learning density for a representative test structure (Fig 4A and 4B). For this test structure, the machine learning density error is ${∊}_{\rho_{ML}}=0.68\%$ with a true error of ${∊}_{\rho_{true}}=1.15\%$, which is comparable to the results for the entire test set in Table 3. The points on the potential surface were constructed from the isovalue of the DNA only density. By varying the isovalue, we sample a range of distances for the potential surface with the variable $\bar{r}$, the average distance of the potential surface to the nearest DNA atom. For reference, the van der Waals radius for hydrogen is $\bar{r}=1.2$ Å, and the distance of a DNA atom to the nearest solvent atom is about $\bar{r}=1.4$ Å. Therefore, the plot samples the electrostatic potential of the solvated DNA test structure inside the first solvation shell.

From the electrostatic potential surfaces, the root-mean-square-deviations (RMSDs) of the machine-learned potential were calculated against the quantum reference potential (Fig 4B), and the averages of the electrostatic potentials were aligned for better comparison. We also compared with the electrostatic potential of the Amber BSC1 classical force field [37, 38], which is parameterized by partial atomic charges, and the potential from a QM/MM calculation, where the DNA atoms were treated quantum mechanically (QM) and the water atoms were replaced with their corresponding TIP3P charges [42]. Throughout the plotted region, the machine-learned potential outperforms both the classical force field and the QM/MM method and shows RMSD values that are stable with the distance $\bar{r}$. The classical force field performs reasonably well though still worse than the machine learning model for distances close to DNA, but performs much worse in the solvent region ($\bar{r}>1.4$ Å). The QM/MM method performs as well as or slightly better than the machine learning model close to DNA, which is unsurprising since QM/MM does not contain any machine learning error for the DNA only. In the solvent region, QM/MM has the same accuracy as the classical force field because QM/MM treats the solvent with the same level of theory. These results show that the machine learning model, which is trained to reproduce a QM level of theory, calculates more accurate electrostatic potentials overall than both a classical force field and QM/MM at a much lower cost than a traditional QM calculation.

To visualize the DNA-solvent interaction, density difference plots were constructed for the QM reference (Fig 4C), machine learning model (Fig 4D), and QM/MM calculation (Fig 4E) using the following steps:

1. The ML model was run on a representative DNA-solvent test structure (Fig 4A) to obtain the electron density for the entire DNA-solvent system.
2. The electron densities of water molecules were removed from the output by zeroing out the atomic basis functions centered on water atoms, giving the “polarized” DNA density *ρ*_*DNA*,*polarized*_.
3. The ML model was subsequently run on the gas-phase DNA version of the same test structure with all water atoms removed to obtain the DNA only density *ρ*_*DNA*,*gas*_.
4. The DNA only density was subtracted from the polarized DNA density, *ρ*_*DNA*,*polarized*_ − *ρ*_*DNA*,*gas*_, to produce a density difference corresponding to local changes in electron density due to solvation. For the QM/MM calculation, this difference is *ρ*_*DNA*,*QM*/*MM*_ − *ρ*_*DNA*,*gas*_, where QM/MM is used for the solvated DNA system.

The plots are presented with the same density isovalue (±0.005 a.u.) for direct comparison, where red indicates an excess of electrons (negative polarization) and blue a deficiency of electrons (positive polarization). Based on its similarity to the quantum reference and the QM/MM difference plots, the machine learning model gets the key features of the DNA-solvent interaction correct. Notably, the DNA shows strong negative polarization around the phosphate groups, which has been shown to be where most of the DNA-solvent charge transfer effects occur [43]. The machine learning density difference plot also appears slightly noisier due to the model prediction error.

### Solvent polarization of a large DNA duplex

Finally, we demonstrate an application for the model on an 18-bp DNA duplex in solution to show that the model can scale up to study systems that are too large for traditional *ab initio* calculations. We selected a DNA structure with a prominent A-tract sequence in its center (PDB code: 1akh) [44], as studies have shown that A-tract sequences have particularly narrow minor grooves that give rise to enhanced negative electrostatic potentials that can be targeted by binding agents [24, 26]. Furthermore, solvent molecules can form a hydration spine along the minor groove that is crucial for non-contact base recognition of sequence-specific binding agents [25].

**Fig 5:**
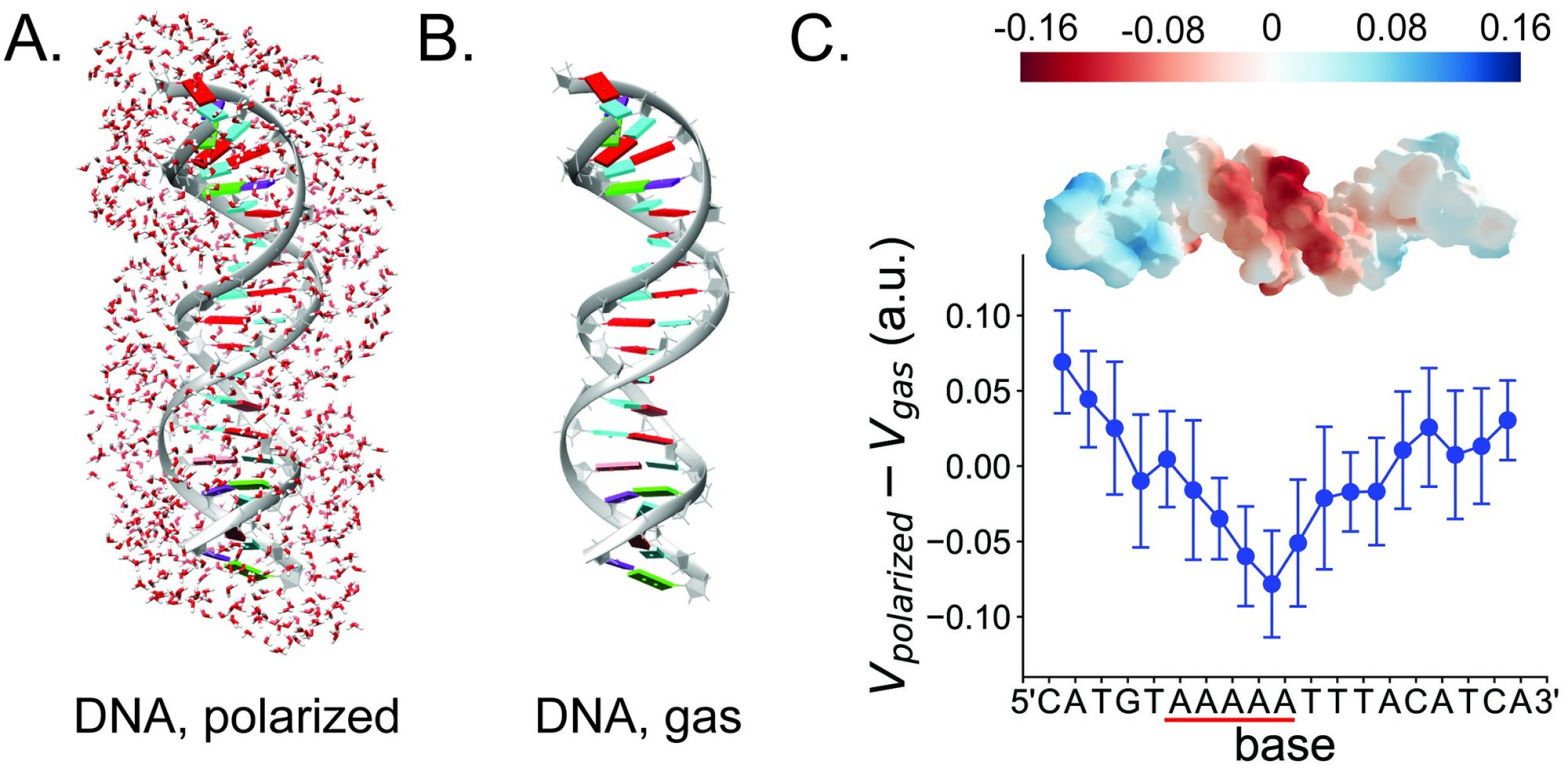
DNA-solvent interaction for a large (18-bp) solvated DNA duplex. (A) DNA with an A-tract sequence in its center (PDB code: 1akh [44]) plus two water shells used to obtain the polarized DNA density. (B) Gas-phase DNA structure of the above. Figures were made with UCSF Chimera [45]. (C) Electrostatic potential difference between the polarized and gas-phase DNA structures on a potential surface (*V*_*DNA*,*polarized*_ − *V*_*DNA*,*gas*_). The surface is calculated for a density isovalue of 0.001 a.u., corresponding to an average distance of about 1.8 Å from the nearest DNA atom. The average of the electrostatic potential difference plot is shifted to zero. The scatter plot shows the electrostatic potential along the DNA axis for the isosurface plot. Points on the potential surface were binned and averaged based on the closest distance to a nucleotide pair of P atoms. The error bars represent the distribution of the potential within a bin. As a guide for the eyes, the scatter plot is roughly aligned with the DNA in the isosurface plot. Electrostatic potential plots were made using Plotly [46].

While a detailed study of DNA solvation, including energetic and dynamic effects, is outside the scope of the paper, we demonstrate that the machine learning model can capture the interaction of a representative static snapshot for a relatively large (18 base pairs) solvated DNA system. To see this effect with our model, we use a similar strategy as in the density difference plots above to visualize the effect of solvent by taking the difference in the electrostatic potentials for the solvent-polarized (Fig 5A) and gas-phase DNA structures (Fig 5B). The solvent-polarized system contains over 4000 atoms, which is well-outside the range of a conventional quantum calculation, and the gas-phase system contains over 1200 atoms. For the gas-phase system, the electrostatic potential is calculated at a density isovalue of 0.001 a.u, corresponding to an average distance on the potential surface of about 1.8 Å from the nearest DNA atom. After taking the difference in the electrostatic potentials of the solvent-polarized and gas-phases DNA systems (*V*_*DNA*,*polarized*_ − *V*_*DNA*,*gas*_), the average of the resulting electrostatic potential is shifted to zero. The resulting difference is plotted in Fig 5C.

Also plotted is cross-sectional information of the electrostatic potential along the DNA axis (Fig 5C). This data was obtained by associating each point on the potential surface to a bin representing a base sequence location based on the point’s closest distance to a nucleotide pair of P atoms. Points within a bin corresponding to a base sequence location are averaged, and the error bars represent the distribution of the potential within a bin. Note that while this distribution is relatively large, the spread is roughly the same size across the DNA axis. The plot shows that for this static snapshot the DNA sequence along the center A-tract is negatively polarized due to the solvent interaction, suggesting the model can capture the physics of the solvent interaction for a relatively large DNA system. Note that in a fully dynamic system, the DNA and solvent configurations and their corresponding interactions will be highly variable due to rapid thermal motion.

## Conclusion

In this study, we successfully extended a machine learning density model previously developed for gas-phase DNA [21, 30] to model solvated DNA with solvent interactions included explicitly. This was achieved by fragmenting the original DNA training structures based on an entire base-pair step into molecular fragments encompassing the key local interactions (i.e. base-pairing, base-stacking, and covalent backbone linkages) as well as the DNA-solvent interactions. From the fragmented training set, we presented three separate models for DNA only, solvent only, and the DNA-solvent interacting systems. The fragment-trained DNA only model unsurprisingly gave higher errors than the model trained on the entire base-pair step structures but still produced highly accurate electron densities, with errors as low as ${∊}_{\rho_{ML}}=0.53\%$. The solvent only model captured polarization effects in water only clusters and around solvated ions that are neglected by classical force fields. Finally, by comparing to reference quantum calculations, the DNA-solvent interacting model resolved the key characteristics of the DNA-solvent interaction and can be scaled up to study solvated DNA systems that are too large for traditional *ab initio* methods. The success of a machine learning density model for DNA-solvent interactions paves the way for future models that can study DNA solvation in detail, including energetic and dynamic effects. Furthermore, the use of smaller fragments in the training set anticipates models trained with larger basis sets that are required to calculate accurate forces for *ab initio* molecular dynamics.

## Computational methods

### Configurational sampling of DNA from all-atom molecular dynamics simulations

Base-pair step configurations are sampled from all-atom molecular dynamics (MD) simulations run with Amber 20 [47] using the BSC1 force field [37, 38]. Given the 4 DNA bases (A, C, G, and T), there are 4^2^ = 16 possible base-pair step sequences. Eliminating redundant sequences due to symmetry from the complementary strand, there are a total of ten unique base pair steps [48, 49]. Ten initial B-DNA 12-mer structures representing the ten unique base-pair steps were each placed in a periodic truncated octahedral box with a 10 Å buffer and solvated with flexible SPC/E water [50]. Note that flexible SPC/E water was used instead of TIP3P water [42] to more realistically sample interacting DNA-solvent geometries. Mg^2+^ counterions were added to neutralize the structure, and then an excess of Mg^2+^ and Cl^-^ at around 100 mmol/L were added to the simulation box. Since only the configurational samples of the central base-pair step of the full 12-mer are used by downstream calculations, harmonic restraints were added to stabilize the base pairs at the DNA ends.

Prior to MD production runs, structures were first minimized then allowed to heat up from 0 K to 300 K for 40 ps. Production runs were performed in the NPT ensemble at 300 K. After heating, 50 ns production runs were performed in the NPT ensemble at 1 atm and 300 K. We used the Langevin thermostat [51, 52] with a collision frequency of 1 ps^−1^ and the Berendsen barostat [53] with a relaxation time of 2 ps. Three separate 20 ns simulations were run with randomized starting trajectories and a time step of 1 fs for a total simulation time of 60 ns. After a buffer time of 5 ns, 300 configurational snapshots were uniformly sampled from each 60 ns trajectory, and fundamental base pair training units were obtained from these snapshots by stripping away all atoms apart from the central two base pairs and the waters required to sample the DNA-solvent interaction. Additionally, we obtain sample structures with Mg^2+^ interacting with the phosphate group by including configurations where the Mg-O atomic distance was less than 2 Å. In addition to this DNA-solvent training set, ionic solvation shells were sampled by extracting water shells around ions outside the influence of the DNA.

### *Ab initio* calculations on the fragmented DNA training set

Density functional theory (DFT) calculations were performed on the fragment training structures to obtain the ground-state electron densities used to train the model. DFT calculations were performed using psi4 [54] with the PBE0 hybrid functional [55] and the aug-cc-pvdz basis [56]. This level of theory was chosen for its balance between computational tractability and accuracy and gave good performance for other density-based machine learning models [17, 21]. In particular, PBE0 is a well-studied hybrid functional and has been shown to give accurate densities close to coupled cluster (CCSD) for its cost [57]. The machine learning model outputs will reflect the level of theory of its training data. In principle, the model can be trained on any level of theory.

Typically, electron densities from *ab initio* calculations are constructed from the wave functions of the occupied states. This representation of the density grows as the square of the system size. To keep the scaling for the machine learning model linear, we project the densities on to an atom-centered “density fitting” basis [58, 59] given by:

$$\begin{array}{c} \rho \left(r\right)=\sum_{i=0}^{N_{atoms}}\sum_{k=0}^{N_{basis}}\sum_{l=0}^{l_{max}}\sum_{m=-l}^{+l}C_{iklm}Y_{lm}e^{-\alpha_{ikl}{\left(r-r_{i}\right)}^{2}}, \end{array}$$ (1)

where the coefficients *α*_*ikl*_ control the Gaussian function widths, *Y*_*lm*_ are the set of spherical harmonic functions, and *C*_*iklm*_ are the set of coefficients for the auxiliary basis. The coefficients *C*_*iklm*_ in the auxiliary density basis are both the outputs of the machine learning model and the data set that the model is trained on. The loss function of the model is calculated by comparing the mean square error of the output coefficients against the training data coefficients. The def2-universal-jfit auxiliary basis was used for this study [59], since expressing the density in this form has been shown to be highly efficient in other machine learning density models [2, 10–12, 17, 21].

For the QM/MM calculation (Fig 4D), the solvent was modeled as an external potential created by replacing water atoms with their corresponding TIP3P point charges [42]. All DNA atoms were treated quantum mechanically with the same settings as in the regular quantum calculations.

### Neural network architecture and parameters

For the sake of neural network training efficiency, it is advantageous to use a machine learning architecture that understands and exploits properties of symmetry such as equivariance. We use the e3nn machine learning framework, which employs a graph convolutional neural network that has equivariance in three dimensions built in [7, 60, 61]. The e3nn framework implements equivariance by representing learned features in the hidden layers of the network as combinations of irreducible representations of 3D space. It has been shown that e3nn can reduce the amount of training data needed by a factor of 1000 compared to models without built-in equivariance [7]. The network we use for this study is built on the gate_points_2101 model, which can be found in the e3nn model library [60].

Detailed descriptions of e3nn and theoretical explorations of Euclidean neural networks can be found elsewhere in the literature [7, 8, 17, 21, 60, 61]. For the purpose of this study, it is sufficient to describe the model inputs and outputs. A schematic of the e3nn training procedure is depicted in Fig 1.

The e3nn model is initialized with a structure’s atomic coordinates. The coordinates are encoded into a three-dimensional graph that gets passed as the input layer to the neural network. Input features are simple one-hot encodings based on element types ([1,0,0,…,0] for an H atom, [0,1,0,…,0] for a C atom, and so on). Nodes in the graph mark atomic centers, and edges represent interactions with nearby atoms. The output of the model is the coefficients *C*_*iklm*_ that represent the 3D charge density in the auxiliary density basis. The adjustable hyperparameters for the neural network were similar to those used in previous e3nn machine learning density studies [17, 21] and are reported in S4 Table.

### Machine learning density prediction error

The machine learning model outputs densities in terms of the auxiliary density basis (def2-universal-jfit [59] in this study). While the output is expressed in terms of the coefficients in this basis, a more physically meaningful measure of the model’s accuracy, the density prediction error ${∊}_{\rho_{ML}}$, can be computed by:

$$\begin{array}{c} \epsilon_{\rho_{ML}}\left(\%\right)=100\times \frac{\int dr|\rho_{ML}\left(r\right)-\rho_{QM_{projected}}\left(r\right)|}{\int dr\rho_{QM_{projected}}\left(r\right)}, \end{array}$$ (2)

where *ρ*_*ML*_(**r**) and $\rho_{QM_{projected}}\left(r\right)$ are the machine learning and quantum mechanical reference densities in the auxiliary basis integrated on a 0.2 Bohr cubic grid. Note that there is an additional contribution to the density error from projecting on to the auxiliary basis. For all tests with the def2-universal-jfit basis, the projection error is constant at around 0.73%. Therefore, the “true” error for the machine learning model is given by:

$$\begin{array}{c} \epsilon_{\rho_{true}}\left(\%\right)=100\times \frac{\int dr|\rho_{ML}\left(r\right)-\rho_{QM_{true}}\left(r\right)|}{\int dr\rho_{QM_{true}}\left(r\right)}, \end{array}$$ (3)

where $\rho_{QM_{true}}$ is the quantum mechanical density in the original orbital basis, not in its projected form. Thus, ${∊}_{\rho_{ML}}$ can be interpreted as the error from fitting the model, and ${∊}_{\rho_{true}}$ adds on the constant error from projecting on to the auxiliary basis. The def2-universal-jfit basis set was used because it is well-studied, has been used in previous machine learning models [2, 10–12, 17, 21], and offers a good balance between basis set size and accuracy.

## Supporting information

S1 Fig Example DNA only fragmented training structures. (A) Base pair fragment, (B) base stacking fragment, and (C) nucleotide fragment.
(PDF)

S2 Fig Example solvent only fragmented training structures. (A) Solvated ion with 15 waters and (B) 15 water only cluster.
(PDF)

S3 Fig Example DNA-solvent fragmented training structures. (A) DNA base with 12 waters and (B) sugar-phosphate backbone with 12 waters and bound Mg^2+^.
(PDF)

S1 Table Contents of the DNA only training set. Combinations 2*10 refer to the two base pair and two base stacking structures in the fundamental base-pair step training unit multiplied by 10 for the possible combinations of base-pair steps. Combinations 4*10 refer to the four base nucleotides (A, C, G, and T) multiplied by 10 for the possible combinations of base-pair steps.
(PDF)

S2 Table Contents of the solvent only training set. (PDF)

S3 Table Contents of the DNA-solvent training set. Combinations 4 refer to the four bases (A, C, G, and T), and combinations 2 refer to structures with and without Mg^2+^ bound to the phosphate.
(PDF)

S4 Table Neural network hyperparameters. Notation example for irreducible representations (irreps): “67x1e” means 67 channels with *l* = 1, even parity. The Adam optimizer was used for the learning rate.
(PDF)

S5 Table Contents of the DNA only model test set for various base sequence lengths. Combinations refer to unique sequences of base pairs for a given sequence length. For 2, 3, and 4 base pairs, combinations are sampled exhaustively. For 5 base pairs, 10 base sequences were generated randomly (S6 Table).
(PDF)

S6 Table Randomly generated base sequences for the DNA only model 5-mer test set. (PDF)

S7 Table Contents of the solvent only model test set. (PDF)

S8 Table Contents of the DNA-solvent model test set. The 11 base pair combinations refer to all 10 possible base-pair step combinations plus a base pair with Mg^2+^ bound to the phosphate.
(PDF)

S9 TableMean signed relative errors ${∊}_{N_{ele}}\left(\%\right)$ for the predicted number of electrons with DNA base sequence length.(PDF)

S1 Appendix Normalizing training data to electron populations. (PDF)
