# Supplementary figures and images for: Building an ab initio solvated DNA model using Euclidean neural networks
Source: PLoS One. 2024 Feb 15;19(2):e0297502. doi: 10.1371/journal.pone.0297502 (PMC10868815; doi:10.1371/journal.pone.0297502)

A.

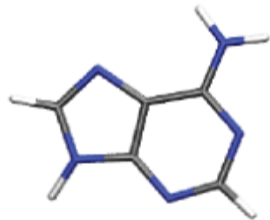

B.

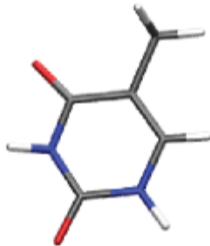

C.

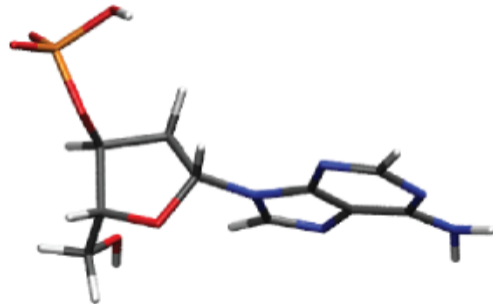

Supplement: S1 Fig — (A) Base pair fragment, (B) base stacking fragment, and (C) nucleotide fragment. (PDF) [file pone.0297502.s001.pdf]

A.

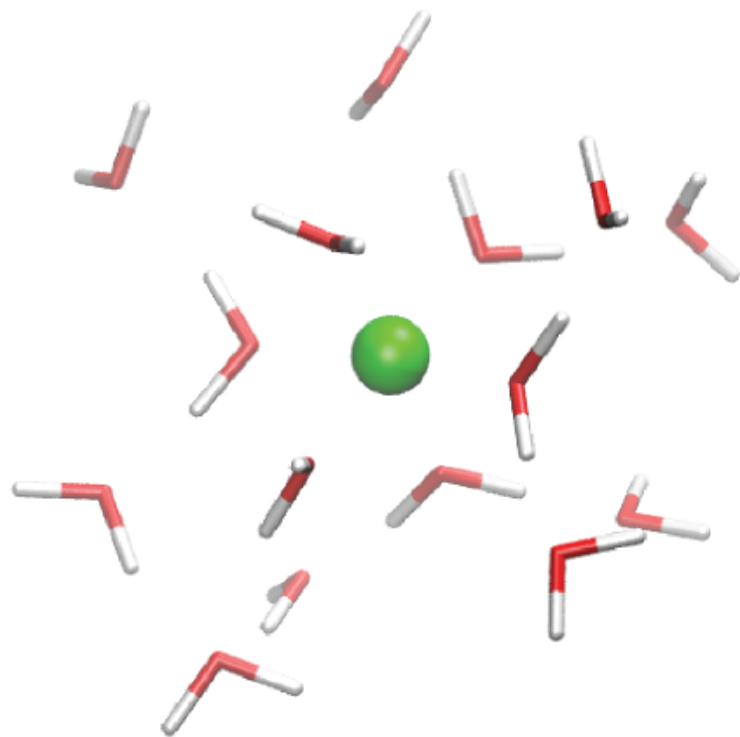

B.

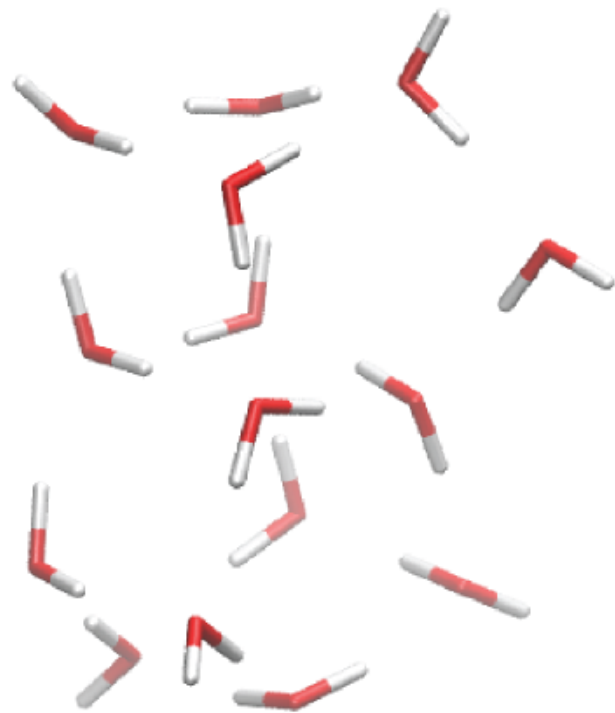

Supplement: S2 Fig — (A) Solvated ion with 15 waters and (B) 15 water only cluster. (PDF) [file pone.0297502.s002.pdf]

A.

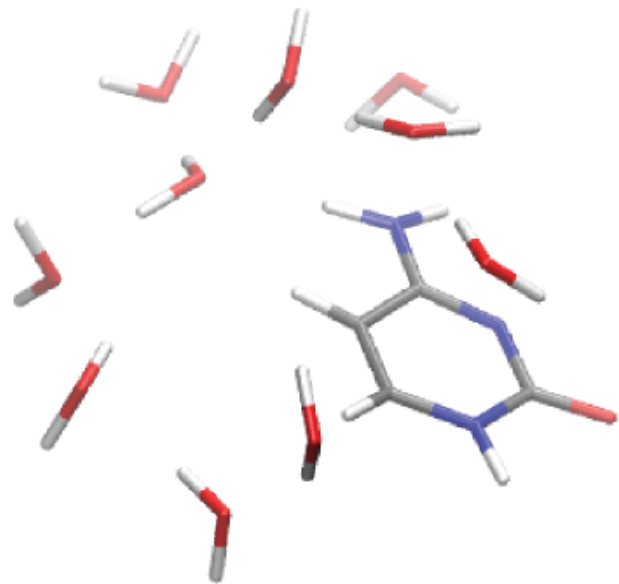

B.

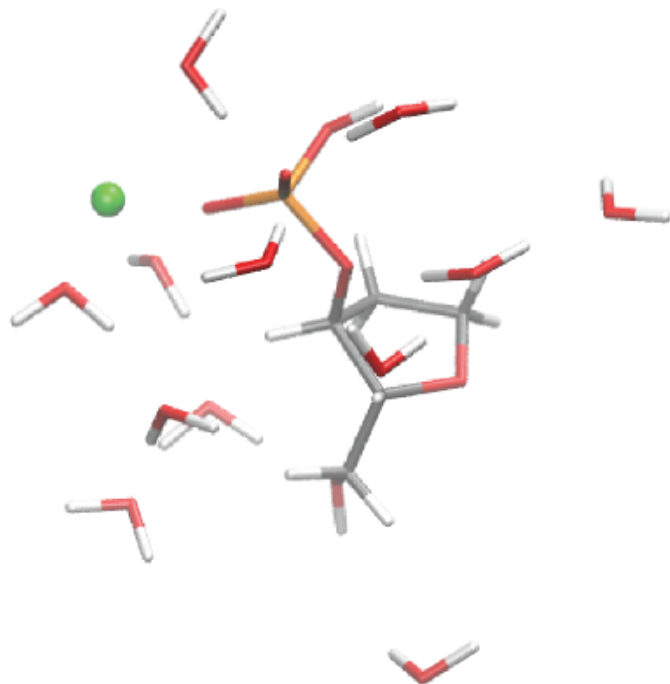

Supplement: S3 Fig — (A) DNA base with 12 waters and (B) sugar-phosphate backbone with 12 waters and bound Mg2+. (PDF) [file pone.0297502.s003.pdf]
