# Supplementary material for: Building an ab initio solvated DNA model using Euclidean neural networks
Source: PLoS One. 2024 Feb 15;19(2):e0297502. doi: 10.1371/journal.pone.0297502 (PMC10868815; doi:10.1371/journal.pone.0297502)
Supplement: S1 Table — Combinations 2*10 refer to the two base pair and two base stacking structures in the fundamental base-pair step training unit multiplied by 10 for the possible combinations of base-pair steps. Combinations 4*10 refer to the four base nucleotides (A, C, G, and T) multiplied by 10 for the possible combinations of base-pair steps. (PDF) [file pone.0297502.s004.pdf]

S1 TABLE. Contents of the DNA only training set. Combinations 2*10 refer to the two base pair and two base stacking structures in the fundamental base pair step training unit multiplied by 10 for the possible combinations of base pair steps. Combinations 4*10 refer to the four base nucleotides (A, C, G, and T) multiplied by 10 for the possible combinations of base pair steps.

**Fragment type**	**Combinations**	**Samples per combination**	**Samples per fragment type**
Base pairs	2*10	200	4000
Base stackings	2*10	200	4000
Nucleotides	4*10	200	8000
**Total samples**			16000
