# Supplementary material for: Building an ab initio solvated DNA model using Euclidean neural networks
Source: PLoS One. 2024 Feb 15;19(2):e0297502. doi: 10.1371/journal.pone.0297502 (PMC10868815; doi:10.1371/journal.pone.0297502)
Supplement: S2 Table — (PDF) [file pone.0297502.s005.pdf]

S2 TABLE. Contents of the solvent only training set.

**Fragment type**	**Number of waters**	**Samples per fragment type**
Solvated Mg^{2+}	15	300
Solvated Cl^{-}	15	300
Water only cluster	15	1000
**Total samples**		1600
