# Supplementary material for: Building an ab initio solvated DNA model using Euclidean neural networks
Source: PLoS One. 2024 Feb 15;19(2):e0297502. doi: 10.1371/journal.pone.0297502 (PMC10868815; doi:10.1371/journal.pone.0297502)
Supplement: S3 Table — Combinations 4 refer to the four bases (A, C, G, and T), and combinations 2 refer to structures with and without Mg2+ bound to the phosphate. (PDF) [file pone.0297502.s006.pdf]

S3 TABLE. Contents of the DNA-solvent training set. Combinations 4 refer to the four bases (A, C, G, and T), and combinations 2 refer to structures with and without Mg^{2+} bound to the phosphate.

**Fragment type**	**Number of waters**	**Combinations**	**Samples per combination**	**Samples per fragment type**
Solvated bases	12	4	300	1200
Solvated sugar-phosphate	12	2	300	600
**Total samples**				1800
