# Supplementary material for: Building an ab initio solvated DNA model using Euclidean neural networks
Source: PLoS One. 2024 Feb 15;19(2):e0297502. doi: 10.1371/journal.pone.0297502 (PMC10868815; doi:10.1371/journal.pone.0297502)
Supplement: S4 Table — Notation example for irreducible representations (irreps): “67x1e” means 67 channels with l = 1, even parity. The Adam optimizer was used for the learning rate. (PDF) [file pone.0297502.s007.pdf]

S4 TABLE. Neural network hyperparameters. Notation example for irreducible representations (irreps): "67x1e" means 67 channels with $l = 1$, even parity. The Adam optimizer was used for the learning rate

`hidden layers:`	5
`irreps hidden:`	200x0e + 200x0o + 67x1e + 67x1o + 40x2e + 40x2o + 29x3e + 29x3o
`irreps hidden` l_{max}`:`	3
`irreps output:`	14x0e + 5x1o + 5x2e + 2x3o + 1x4e
`radial max radius:`	4.0 Å
`radial basis functions:`	10
`radial layers:`	1
`radial neurons:`	128
`number of epochs:`	350
`learning rate:`	0.01 and 0.001
