# Supplementary material for: Building an ab initio solvated DNA model using Euclidean neural networks
Source: PLoS One. 2024 Feb 15;19(2):e0297502. doi: 10.1371/journal.pone.0297502 (PMC10868815; doi:10.1371/journal.pone.0297502)
Supplement: S5 Table — Combinations refer to unique sequences of base pairs for a given sequence length. For 2, 3, and 4 base pairs, combinations are sampled exhaustively. For 5 base pairs, 10 base sequences were generated randomly (S6 Table). (PDF) [file pone.0297502.s008.pdf]

S5 TABLE. Contents of the DNA only model test set for various base sequence lengths. Combinations refer to unique sequences of base pairs for a given sequence length. For 2, 3, and 4 base pairs, combinations are sampled exhaustively. For 5 base pairs, 10 base sequences were generated randomly (Table S6 TABLE.).

Sequence length	Base pair combinations	Samples per combination	Total samples
2	10	30	300
3	32	10	320
4	136	2	272
5	10	3	30
