# Supplementary material for: Building an ab initio solvated DNA model using Euclidean neural networks
Source: PLoS One. 2024 Feb 15;19(2):e0297502. doi: 10.1371/journal.pone.0297502 (PMC10868815; doi:10.1371/journal.pone.0297502)
Supplement: S6 Table — (PDF) [file pone.0297502.s009.pdf]

S6 TABLE. **Randomly generated base sequences for the DNA only model 5-mer test set.**

label	base sequence
1	GTACT
2	CCACC
3	AACCT
4	ATCGA
5	AAGGG
6	CACCG
7	CTTGC
8	AAGCT
9	ATTAA
10	GCGGA
