# Supplementary material for: Building an ab initio solvated DNA model using Euclidean neural networks
Source: PLoS One. 2024 Feb 15;19(2):e0297502. doi: 10.1371/journal.pone.0297502 (PMC10868815; doi:10.1371/journal.pone.0297502)
Supplement: S7 Table — (PDF) [file pone.0297502.s010.pdf]

S7 TABLE. Contents of the solvent only model test set.

Type	Number of waters	Total samples
Solvated Mg^{2+}	20	10
Solvated Cl^{-}	20	10
Water only cluster	20	10
