# Supplementary material for: Building an ab initio solvated DNA model using Euclidean neural networks
Source: PLoS One. 2024 Feb 15;19(2):e0297502. doi: 10.1371/journal.pone.0297502 (PMC10868815; doi:10.1371/journal.pone.0297502)
Supplement: S8 Table — The 11 base pair combinations refer to all 10 possible base-pair step combinations plus a base pair with Mg2+ bound to the phosphate. (PDF) [file pone.0297502.s011.pdf]

S8 TABLE. Contents of the DNA-solvent model test set. The 11 base pair combinations refer to all 10 possible base pair step combinations plus a base pair with Mg^{2+} bound to the phosphate.

Sequence length	Number of waters	Base pair combinations	Samples per combination	Total samples
2	100	11	10	110
