# Supplementary material for: Building an ab initio solvated DNA model using Euclidean neural networks
Source: PLoS One. 2024 Feb 15;19(2):e0297502. doi: 10.1371/journal.pone.0297502 (PMC10868815; doi:10.1371/journal.pone.0297502)
Supplement: S9 Table — (PDF) [file pone.0297502.s012.pdf]

S9 TABLE. **Mean signed relative errors $\epsilon_{N_{ele}}(\%)$ for the predicted number of electrons with DNA base sequence length.**

Model	$\epsilon_{N_{ele}}(\%)$ per base sequence length			
	**2**	**3**	**4**	**5**
entire base pair step	0.0036 ± 0.028	-0.015 ± 0.027	-0.027 ± 0.026	-0.033 ± 0.020
fragmented	-0.020 ± 0.049	-0.084 ± 0.044	-0.10 ± 0.044	-0.11 ± 0.036
