# Supplementary material for: Building an ab initio solvated DNA model using Euclidean neural networks
Source: PLoS One. 2024 Feb 15;19(2):e0297502. doi: 10.1371/journal.pone.0297502 (PMC10868815; doi:10.1371/journal.pone.0297502)
Supplement: S1 Appendix — (PDF) [file pone.0297502.s013.pdf]

S1 APPENDIX. Normalizing training data to electron populations.

We use two techniques to normalize the training data for more efficient learning. First, coefficients in the output density basis are normalized to be populations. This means that a coefficient for an $l = 0$ function corresponds to the number of electrons contained in that function. Note that for $l > 0$ functions, the number of electrons contained will always be 0 because the positive and negative components of the function cancel out. Therefore, we normalize the absolute value of the $l > 0$ functions to be an electron population. In previous studies with the model, we normalized all functions based on the $l = 0$ form, causing some coefficients for higher l functions to be more sensitive to machine learning error. The scheme presented here normalizes the sensitivity and leads to more efficient learning and lower errors in density predictions. Comparisons to these previous models in the paper were rerun based off of the improved normalization scheme.

The second technique is to subtract the $l = 0$ functions of isolated atoms (obtained from running quantum calculations on isolated H, isolated C, and so on) from the output coefficients. Because the functions were normalized to electron populations, for a neutral system, the sum of the $l = 0$ coefficients should be 0. In this way, the $l = 0$ coefficients are rescaled and do not dominate the loss function.
